# Effect of Amine Functionalization of MOF Adsorbents for Enhanced CO_2_ Capture and Separation: A Molecular Simulation Study

**DOI:** 10.3389/fchem.2020.574622

**Published:** 2021-01-11

**Authors:** Daniel Bahamon, Wei Anlu, Santiago Builes, Maryam Khaleel, Lourdes F. Vega

**Affiliations:** ^1^Chemical Engineering Department, Research and Innovation Center on CO_2_ and H_2_ (RICH), Khalifa University, Abu Dhabi, United Arab Emirates; ^2^Center for Catalysis and Separation (CeCaS), Khalifa University, Abu Dhabi, United Arab Emirates; ^3^Chemical Engineering Department, China University of Petroleum, Dongying, China; ^4^Process Engineering Department, EAFIT University, Medellin, Colombia

**Keywords:** CO_2_ capture, metal-organic frameworks, MOF-74, amine, functionalization, Monte Carlo simulation, chemisorption

## Abstract

Different types of amine-functionalized MOF structures were analyzed in this work using molecular simulations in order to determine their potential for post-combustion carbon dioxide capture and separation. Six amine models -of different chain lengths and degree of substitution- grafted to the unsaturated metal sites of the M_2_(dobdc) MOF [and its expanded version, M_2_(dobpdc)] were evaluated, in terms of adsorption isotherms, selectivity, cyclic working capacity and regenerability. Good agreement between simulation results and available experimental data was obtained. Moreover, results show two potential structures with high cyclic working capacities if used for Temperature Swing Adsorption processes: mmen/Mg/DOBPDC and mda-Zn/DOBPDC. Among them, the -mmen functionalized structure has higher CO_2_ uptake and better cyclability (regenerability) for the flue gas mixtures and conditions studied. Furthermore, it is shown that more amine functional groups grafted on the MOFs and/or full functionalization of the metal centers do not lead to better CO_2_ separation capabilities due to steric hindrances. In addition, multiple alkyl groups bonded to the amino group yield a shift in the step-like adsorption isotherms in the larger pore structures, at a given temperature. Our calculations shed light on how functionalization can enhance gas adsorption *via* the cooperative chemi-physisorption mechanism of these materials, and how the materials can be tuned for desired adsorption characteristics.

## Introduction

Rapid economic growth and continued industrial development have led to an increase of carbon dioxide in the atmosphere (Spigarelli and Kawatra, 2013). Hence, mitigation strategies such as Carbon Capture, Utilization, and Storage (CCUS) play an important role to limit the contribution of these emissions to the global climate change (IPCC, 2014). CO_2_ post-combustion capture from flue gas at power stations and chemical manufacturing plants is one of the key approaches for reducing these emissions (D'Alessandro et al., 2010). The concentration of CO_2_ in the flue gas produced by combustion is around 5–15%, under atmospheric pressure and at temperatures between 30 and 60°C (D'Alessandro et al., 2010; Sumida et al., 2011). The remaining components of the flue gas are mainly N_2_ and a small amount of secondary components including O_2_, H_2_O, CO, SOx, and NOx. In most cases the exhaust stream is treated before entering the CCUS system to reduce the concentration of these secondary species in the flue gas, as they might significantly affect the operation, even if present in trace concentrations (Spigarelli and Kawatra, 2013).

Absorption with aqueous amine solutions (e.g., monoethanolamine, MEA) is conventionally used to capture CO_2_ at large scale (Bourzac, 2017; Rochelle, 2009). The low solvent cost and good trapping effect make it the most popular and developed carbon capture technology. This strategy has a wide range of applications and can be applied to capture CO_2_ from streams with low concentrations. However, it presents several disadvantages such as high-energy consumption required for regeneration, degradation in the presence of oxygenated compounds, loss of amines by evaporation, and corrosion (Sumida et al., 2011; Huck et al., 2014; Alkhatib et al., 2020).

Alternatively, solid adsorbents are becoming popular aimed at improving the shortcomings of amine solutions. As such, the use of adsorbents has been proposed as an alternative for CO_2_ capture (Hedin et al., 2010; Mukherjee et al., 2019). Adsorbents can exhibit high affinity for selective CO_2_ adsorption under flue gas conditions. In this regard, zeolites, activated carbons, silicas, porous organic networks, and metal–organic frameworks (MOFs), among others, may offer enhanced stability, greater CO_2_ cycling capacities, and inherently lower regeneration energies (Choi et al., 2009; Bae and Snurr, 2011; Bollini et al., 2011; Sumida et al., 2011; Huck et al., 2014; Webley, 2014; Bui et al., 2018; Bahamon et al., 2019; Siegelman et al., 2019).

Among them, MOFs are a class of porous crystalline materials consisting of metal nodes connected by polytopic organic linkers, bearing high surface areas and highly tunable pore characteristics (Eddaoudi et al., 2002; Zhou et al., 2012; Furukawa et al., 2013). Research interest in MOFs has grown dramatically in the past decade, largely driven by their potential in gas storage, separation, catalysis, sensing, and drug delivery, to name a few (Eddaoudi et al., 2002; Lee et al., 2009; D'Alessandro et al., 2010; Keskin et al., 2010; McKinlay et al., 2010; Bae and Snurr, 2011; Mason et al., 2011; Shah et al., 2012). MOFs with high porosity, open metal uncoordinated sites and good thermal stability have demonstrated excellent qualities for CO_2_/N_2_ separations (Yazaydin et al., 2009; Zhang et al., 2010; Sumida et al., 2011; Yu et al., 2017) and are emerging as promising candidates for CO_2_ separation at industrial conditions. For instance, the M-MOF-74 family (with M being the divalent metal used), also known as M_2_(dobdc) or CPO27-M, presents one of the highest found CO_2_ adsorption capacities at low to moderate CO_2_ partial pressures, relevant at the required conditions for CO_2_ capture from flue gas (Britt et al., 2008; Caskey et al., 2008; Dietzel et al., 2009; Yazaydin et al., 2009; Valenzano et al., 2010; Alonso et al., 2018; Bahamon et al., 2020). However, in some cases, because CO_2_ typically adsorbs via weak physisorption interactions, most of these synthesized structures cannot satisfy industrial requirements. MOFs usually exhibit moderate CO_2_/N_2_ selectivity at low CO_2_ partial pressures, and are unable to remove most of the CO_2_ captured in the regeneration step (Vega and Bahamon, 2016). Furthermore, the use of the most promising MOFs require partial or complete drying of the gas stream because high affinity for CO_2_ entails hydrophilicity, and thus water can competitively adsorb with CO_2_ (Keskin et al., 2010; Sayari et al., 2011; Bahamon et al., 2018).

Therefore, amine-functionalized MOFs are attracting great attention, as they are more effective than the corresponding original solid adsorbents (McDonald et al., 2012; Planas et al., 2013; Qiao et al., 2016). In fact, functionalization is recognized today as an effective technique to improve the adsorption and separation of CO_2_ by MOFs and other adsorbent materials. Studies have shown that amine-functionalized MOFs have potential for greater adsorption capacity, higher selectivity, faster CO_2_ adsorption kinetics, and lower regeneration temperatures, making them good candidates for process adsorbents (Arstad et al., 2008; Vaidhyanathan et al., 2010; Choi et al., 2012; Das et al., 2012; Chen et al., 2013; Wang et al., 2013; Bernini et al., 2015; McDonald et al., 2015; Fracaroli et al., 2016; Huang et al., 2016; Liao et al., 2016; Lin et al., 2016; Qiao et al., 2016; Kang et al., 2019). For instance, by grafting ethylenediamine (-en) onto the open metal sites, Choi et al. (2012) modified Mg-MOF-74 and found that both CO_2_ adsorption capacity and the regenerability of the material were enhanced, especially in the lower pressure range. The secondary amine N,N'-dimethylethylenediamine (-mmen) was grafted onto the exposed Cu^2+^ sites of the Cu-BTTri framework, ensuing in a significantly enhanced CO_2_ capacity at low concentrations, related to the formation of zwitterionic carbamate or carbamic acid (Lee et al., 2014). Ultrahigh CO_2_/N_2_ selectivity was observed for polyethyleneimine (PEI)-impregnated MIL-101 (up to 1,200 at 50°C), alkylamine-tethered MIL-101 (up to 346), mmen-Cu-BTTri (327 under post-combustion conditions) and diamine-grafted Mg_2_(dobpdc) (dobpdc=4,4'-dihydroxy-(1,10-biphenyl)-3,3'-dicarboxylic acid) (up to 230) (McDonald et al., 2012; Lin et al., 2013; Lee et al., 2014).

Recently, functionalization with alkyldiamines on the unsaturated metal sites of M_2_(dobpdc) (herein after called M/DOBPDC), an expanded version of the well-studied MOF-74, have demonstrated to be a simple methodology for increasing low pressure CO_2_ adsorption selectivity and capacity (Demessence et al., 2009; Lee et al., 2014; McDonald et al., 2015; Jo et al., 2016; Milner et al., 2017, 2018; Kang et al., 2019). Formation of ammonium carbamate chains takes place for different diamine molecules on the adsorbent, leading to isotherms exhibiting step-like shapes at given temperatures (Choi et al., 2012; McDonald et al., 2015; Vlaisavljevich et al., 2015; Yeon et al., 2015; Jo et al., 2016; Milner et al., 2017, 2018; Siegelman et al., 2017; Forse et al., 2018; Lee et al., 2018). This type of material has been tested experimentally to be stable for at least 1,000 adsorption–desorption cycles (Forse et al., 2018), and this step-like adsorption can be tuned to meet different specifications. Moreover, functionalized M/DOBPDC materials showed greater stability than the non-functionalized counterparts when exposed to humidity and atmospheric conditions (McDonald et al., 2015; Siegelman et al., 2017).

Although recent achievements in post-functionalization have partially revealed the underlying CO_2_ adsorption mechanism for some materials, the optimal (i) MOF/amine combinations, and (ii) number of amine functional groups required for optimizing the performance of such materials for real-world CO_2_ capture applications, remain elusive (Kang et al., 2019). Hence, it is of great significance to understand how functionalized MOFs can be rationally designed from a computational approach to guide their efficient synthesis (Qiao et al., 2016). As molecular simulations allow the systematic and precise study of the various relevant variables of the system, they allow to isolate and quantify the effect of each of them on the performance of the system (Bahamon and Vega, 2019), being an excellent tool for the rational design of materials. Therefore, molecular simulations were used in this contribution to explore the relationship between the structure of MOFs and their CO_2_ adsorption performance when amino-functionalized. A series of amine-grafted MOF-74, and the expanded version M/DOBPDC, were screened, establishing the most promising materials for adsorbing low-concentration CO_2_, while considering their regeneration performance (cyclability). This computational study offers a molecular understanding on how the functionalization takes place on such materials and how it affects their final performance, providing guidance on the design of the best material/amine combination for optimal post-combustion CO_2_ capture.

## Methodology

### Adsorbent Structures

M-MOF-74 possess a high density of exposed M^2+^ (M = Mg, Zn, Mn, Co, etc; dobdc^4−^ = 4,6-dioxido-1,3-benzenedicarboxylate) sites (Rowsell and Yaghi, 2006). This family-type of MOFs has a honeycomb network topology, and the one-dimensional hexagonal channels are covered with bare metal ion adsorption sites (Pentyala et al., 2016). Such bare metal sites are the preferred location for adsorption of CO_2_ molecules at lower pressures. The coordinatively unsaturated M^2+^ sites in these materials lead to superior performance for the physisorptive separation of CO_2_ compared to other MOFs (Caskey et al., 2008; Alonso et al., 2018). Moreover, such unsaturated sites can be occupied by amines via coordination for improving the adsorption capabilities (Choi et al., 2012).

**Figure 1:**
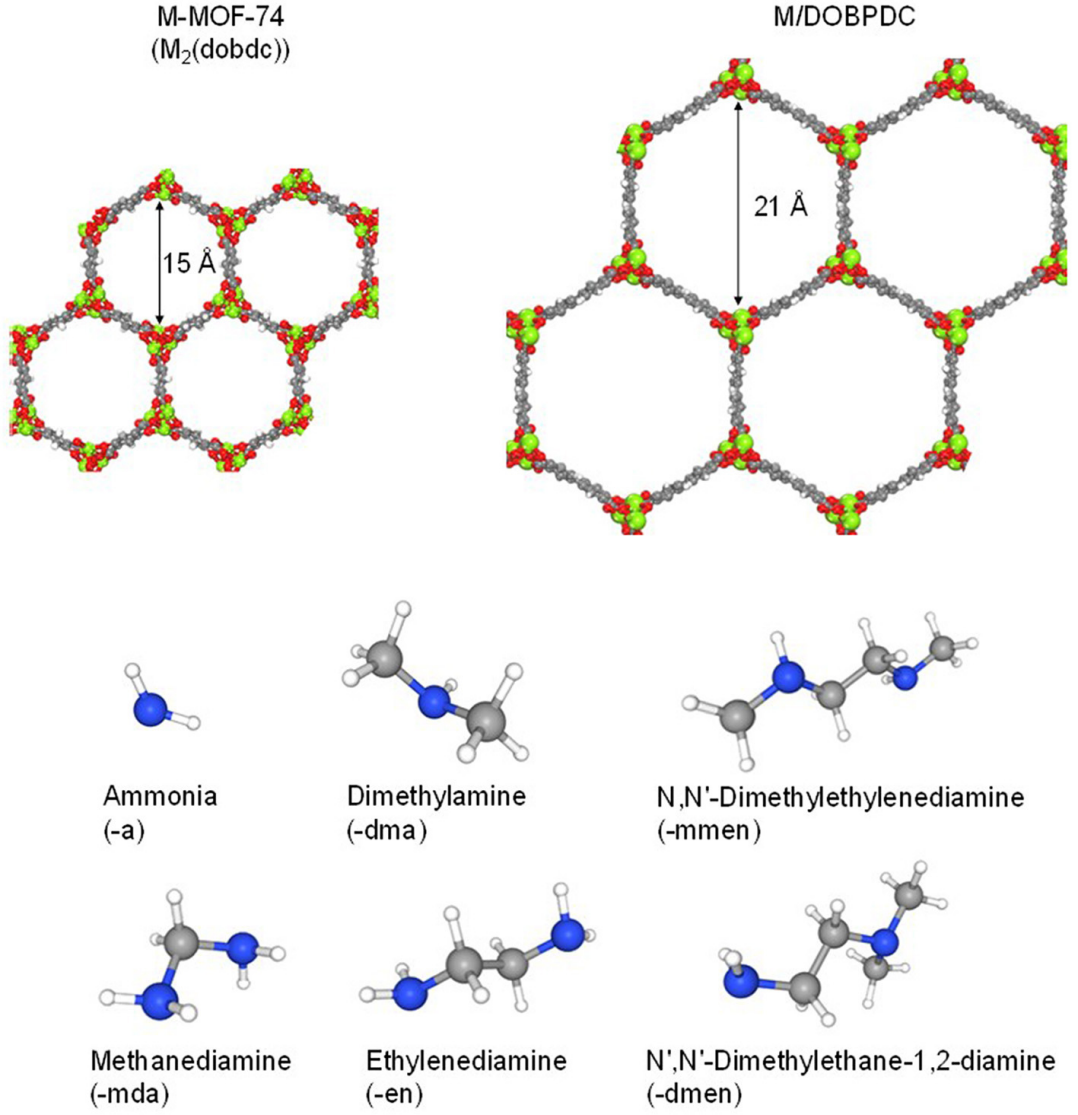
Molecular representation of the MOF structures studied in this work, and amine molecules used for functionalization [Color code: C, H, O, N, Mg (metal) in gray, white, red, blue, and green, respectively].

Expanded versions of materials belonging to the MOF-74 family have been synthesized using longer linker analogues, providing materials with extremely high porosity (Deng et al., 2012, Yeon et al., 2015; Forse et al., 2018). For instance, Mg/DOBPDC has a surface area (S_BET_) of 2,451 m^2^·g^−1^ greater than the 816 m^2^·g^−1^ for Mg-MOF-74 (McDonald et al., 2012). Therefore, the expanded form, with average pore diameter of 21Å (McDonald et al., 2012), was also chosen to be studied in this contribution. The enlarged pores provide enough space to accommodate longer alkylamines onto the open metal sites of the framework compared to M-MOF-74 materials. Hence, in this work, the open metal sites in the microporous channels were functionalized with different amines, including primary amines ethylenediamine (-en) and methanediamine (-mda); secondary amine N,N′-dimethylethylenediamine (-mmen); while 1,1-dimethylethylenediamine (-dmen) and dimethylamine (dma) were evaluated as ternary amines. Also, ammonia [(-a) as abbreviation] was included as a functionalization moiety. Crystallographic information for the solid-state structures (without and with amine-grafted molecules) can be found elsewhere (Choi et al., 2012; Milner et al., 2017; Siegelman et al., 2017; Lee et al., 2018). Structures of the different materials studied in this work are shown in Figure 1. The focus of this contribution is to gain understanding of the CO_2_ adsorption performance depending on the channel size and amine groups appended onto the metal sites. Moreover, different degrees of functionalization have been included by molecular simulations (e.g., 100% functionalization means one amine grafted molecule bounded to each metal center of the crystallographic structure) as part of the study, in order to quantify the effect of the functionalization degree on the adsorption behavior.

### Simulation Details and Calculated Parameters

Grand Canonical Monte Carlo (GCMC, i.e., at constant chemical potential, volume and temperature) simulations were used to obtain adsorption properties for pressures ranging from 1 to 140 kPa. All simulations were performed using the LAMMPS code (Plimpton, 1995). Monte Carlo moves/steps were done with equal probability for translation, rotation, insertion, deletion, and random reinsertion of an existing molecule at a new position. The average number of adsorbed molecules of the system was calculated sequentially at each pressure point, to construct the adsorption isotherm. 1 × 10^6^ MC equilibration steps and 1 × 10^6^ MC production steps were used for each pressure point. Pure CO_2_ as well as a binary mixture containing 15% CO_2_ and 85% N_2_ (based on typical flue gas compositions) were simulated. Water and other impurities were omitted as multi-component mixtures are out of the scope of this study.

Molecular models of CO_2_ and N_2_ molecules were taken from TraPPE (Potoff and Siepmann, 2001) forcefield, which uses a rigid geometry considering only non-bonded interactions. Dzubak and Mercado force fields were used to account for the parameters of the MOFs (Dzubak et al., 2012; Mercado et al., 2016), while Lennard Jones (LJ) and Coulomb parameters of C, H, and N atoms belonging to the amine molecules were taken from the extension of TraPPE force field for nitrogen containing molecules (Wick et al., 2005). Moreover, the paired interactions between the framework atoms were excluded since the structures were considered frozen (including the amine-grafted molecules) to save computational time. The simulation cell was replicated to create 2x2x4 supercell (dimensions of ~ 40 × 40 × 28 Å). In each simulation step, the total energy calculation of the system was computed as the sum of the interaction energies of the adsorbate-adsorbent material and the adsorbate-adsorbate molecules. Lorentz–Berthelot standard mixing rules were applied for all cross terms, and Lennard-Jones interactions beyond 12.8 Å were neglected. The electrostatic interactions were computed with a relative accuracy of 10^−5^ by means of the Ewald summation method.

Since it is known that the amine groups chemically react with CO_2_ and enable adsorption enhancement at very low pressures, chemisorption was explicitly considered as proposed by Builes and Vega (2012, 2013), Builes et al. (2015). According to previous reports in the literature (Lee et al., 2014; Siegelman et al., 2017), CO_2_ molecules are inserted into the metal-amine bonds, promoting a reorganization of the amines into well-ordered chains. The interaction between the amines and CO_2_ in the pores leads to the formation of carbamic acid, as previously documented from DFT calculations (Lee et al., 2014). Therefore, in this work, instead of randomly binding selected metal centers first by the functional groups (either amine or carbamate), and then growing the rest of the amine molecule using a configurational bias algorithm (Builes and Vega, 2012), we started from a configuration extracted from reported crystallographic data files (Choi et al., 2012; Milner et al., 2017; Siegelman et al., 2017; Lee et al., 2018), where carbamic acid formation was already present. Additional CO_2_ physisorption was then evaluated starting from this configuration, for different degrees of functionalization. Furthermore, we considered that the metal centers of the MOFs only bind with carbon dioxide and then these with the amine groups, and no further amine-bridges or polymerization were formed between additional neighboring groups than 100% functionalization.

In mixtures, one additional indicator for determining the separation capacity of porous adsorbent is its adsorption selectivity. Selectivity was calculated as:

(1) $$\begin{array}{r} s_{CO_{2}/N_{2}}=\frac{\left(\frac{x_{CO_{2}}}{x_{N_{2}}}\right)}{\left(\frac{y_{CO_{2}}}{y_{N_{2}}}\right)} \end{array}$$

where *x* represents the molar fraction in the adsorbed phase and *y* the molar fraction in the bulk phase (i.e., feed conditions). The selectivity values presented in this work were directly calculated from the GCMC simulations of the mixtures, rather than from the pure isotherm data published in the theoretical and experimental studies.

For process evaluation, a particularly important attribute frequently used as an evaluation criterion in the adsorption process is the cyclic working capacity. The calculation method is:

(2) $$\begin{array}{r} \Delta N_{CO_{2}}=N_{CO_{2}}^{mix}\left(P_{ads},T_{ads},y\right)-N_{CO_{2}}^{mix^{\prime }}\left(P_{des},T_{des},y\right) \end{array}$$

where $N_{CO_{2}}^{mix}\left(P_{ads},T_{ads},y\right)$ and $N_{CO_{2}}^{mix^{\prime }}\left(P_{des},T_{des},y\right)$ stand for the uptake per mass of adsorbent under adsorption (feed) and desorption (regeneration) conditions, respectively, both involving pressure, temperature and mixture composition. For materials highly selective to CO_2_, a good assumption is that pure CO_2_ is recovered at the outlet of the adsorber (Vega and Bahamon, 2016; Prats et al., 2017). Usually this working capacity is more important than the total absorbed capacity because it defines the amount that can be recovered in each adsorption cycle. Moreover, regenerability is defined as the fraction of the bed used to collect the captured CO_2_, and was calculated as:

(3) $$\begin{array}{r} Regenerability=\frac{\Delta N_{CO_{2}}}{N_{CO_{2}}^{mix}\left(P_{ads},T_{ads},y\right)} \end{array}$$

## Results and Discussion

### Validation and Comparison of Pure CO_2_ Isotherms

**Figure 2:**
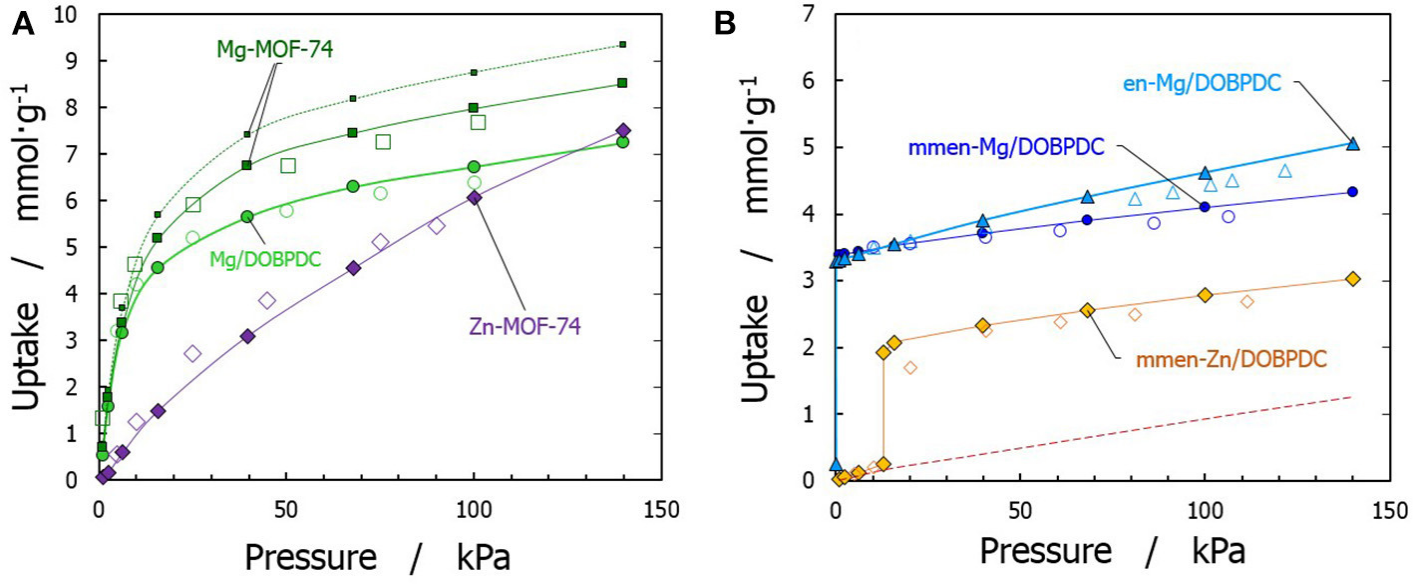
Validation of simulated (filled symbols) adsorption isotherms of carbon dioxide with available experimental data (open symbols) for **(A)** bare materials [squares from Mason et al. (2011), at 313 K; circles from McDonald et al. (2015), at 298 K; diamonds from Caskey et al. (2008), at 296 K], and **(B)** functionalized materials [triangles from Lee et al. (2014), at 298 K; circles and diamonds from McDonald et al. (2015), at 313 K]. Lines are to guide the eyes. For bare Mg-MOF-74, dotted line represents pristine material and straight line is the isotherm after using a scaling factor according to Dzubak et al. (2012).

To confirm the reliability of the force fields adopted in this work, the simulated adsorption isotherms of pure CO_2_ were compared with experimental data at their corresponding temperatures (Caskey et al., 2008; Mason et al., 2011; Lee et al., 2014; McDonald et al., 2015). Compared materials include amine-functionalized en-Mg/DOBPDC, mmen-Mg/DOBPDC, and mmen-Zn/DOBPDC, as well as the bare Mg-MOF-74, Zn-MOF-74, and Mg/DOBPDC frameworks (see Figure 2). A good agreement with the corresponding experimental data was obtained for the amine-grafted materials after using the explicit chemisorption technique (Builes and Vega, 2012; Builes et al., 2015). However, it should be mentioned that a discrepancy of ca. 10% is presented for bare Mg-MOF-74 between the experimental and simulated isotherm, due to the use of a perfect crystal (i.e., no defects and no solvent impurities affecting accessibility for all the Mg sites), as previously explained by Dzubak et al. (2012). This difference is diminished when using a scaling factor for the simulated isotherm. Moreover, note that such treatment has more effect in this bare structure, since highly coordinated Mg-CO_2_ interactions are dominant at low pressures. Nevertheless, the effect is expected to show a minor contribution in the functionalized structures, since the metal sites are already occupied by a chemisorbed CO_2_ molecule and a bulky amine. For the other eleven studied materials, to the best of our knowledge, there are no experimental data available. Yet, we feel confident that the simulations enable good reproduction of the experimental process, as the validated structures/molecules are representative of the additional functional groups studied.

At this point, is important to mention that most of the structures that have been experimentally explored use Magnesium as the open metal site (Lee et al., 2018; Kang et al., 2019). Therefore, stronger interactions are achieved but the regeneration will be more difficult because the adsorption uptake can still be significant at high temperatures. Therefore, simulated structures using a less attractive metal, such as Zinc, were also performed, as it is expected that the steepness of the isotherms gradually diminish as the temperature increases in these materials.

**Figure 3:**
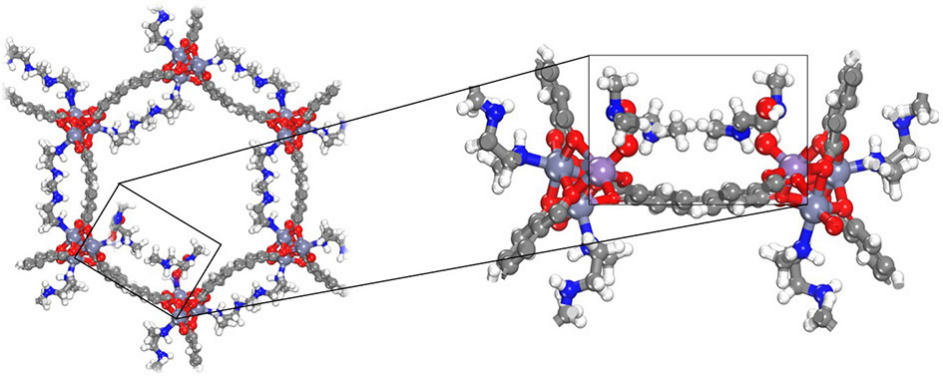
Three-dimensional structure representation of carbamic acid functionalization for mmen-Zn/DOBPDC (Color code: C, H, O, N, Zn in gray, white, red, blue, and purple, respectively].

Figure 2 also shows the obtained CO_2_ adsorption isotherm for mmen-Zn/DOBPDC without considering the chemisorption process (red dashed line). As previously mentioned, the isotherms of amine grafted M/DOBPDC materials display step-like adsorption behavior (sigmoidal) at low pressures (McDonald et al., 2015), corresponding to strong interactions of CO_2_ with the frameworks and the amine moiety. The simulations show that this step originates from a mechanism wherein the CO_2_ gas molecule is cooperatively and reversibly inserted into metal–amine bond followed by formation of ammonium carbamate chain structure (Lee et al., 2018), as shown in Figure 3. When this critical step is reached and chemisorption occurs, then adsorption gradually increases with pressure due to the physisorption phenomenon. Such critical uptake was found to be 0.25 mmol·g^−1^ for the mmen-Zn/DOBPDC structure validated (i.e., one CO_2_ molecule in a crystal with 18 metal centers) and was assumed to be similar for the other hypothetical similar structures evaluated. Nevertheless, it should be mentioned that such critical point cannot be generalized to other metal centers and structures, because is highly dependent on the specific surface area (Builes et al., 2015) and the solid-fluid interactions. In fact, the assumption of both frozen frameworks and grafted-amine molecules appears as a good approximation for the pressure range studied, where adsorbents saturation has not been reached and steric effects can have a less important influence. This was validated by the agreement with experimental data at these conditions. However, the reader must be aware that enthalpic and entropic contributions when moving to higher pressures and temperatures must be considered, in this case, allowing the amines to move and reach their relaxed energetic position inside the MOF (Builes and Vega, 2012).

**Figure 4:**
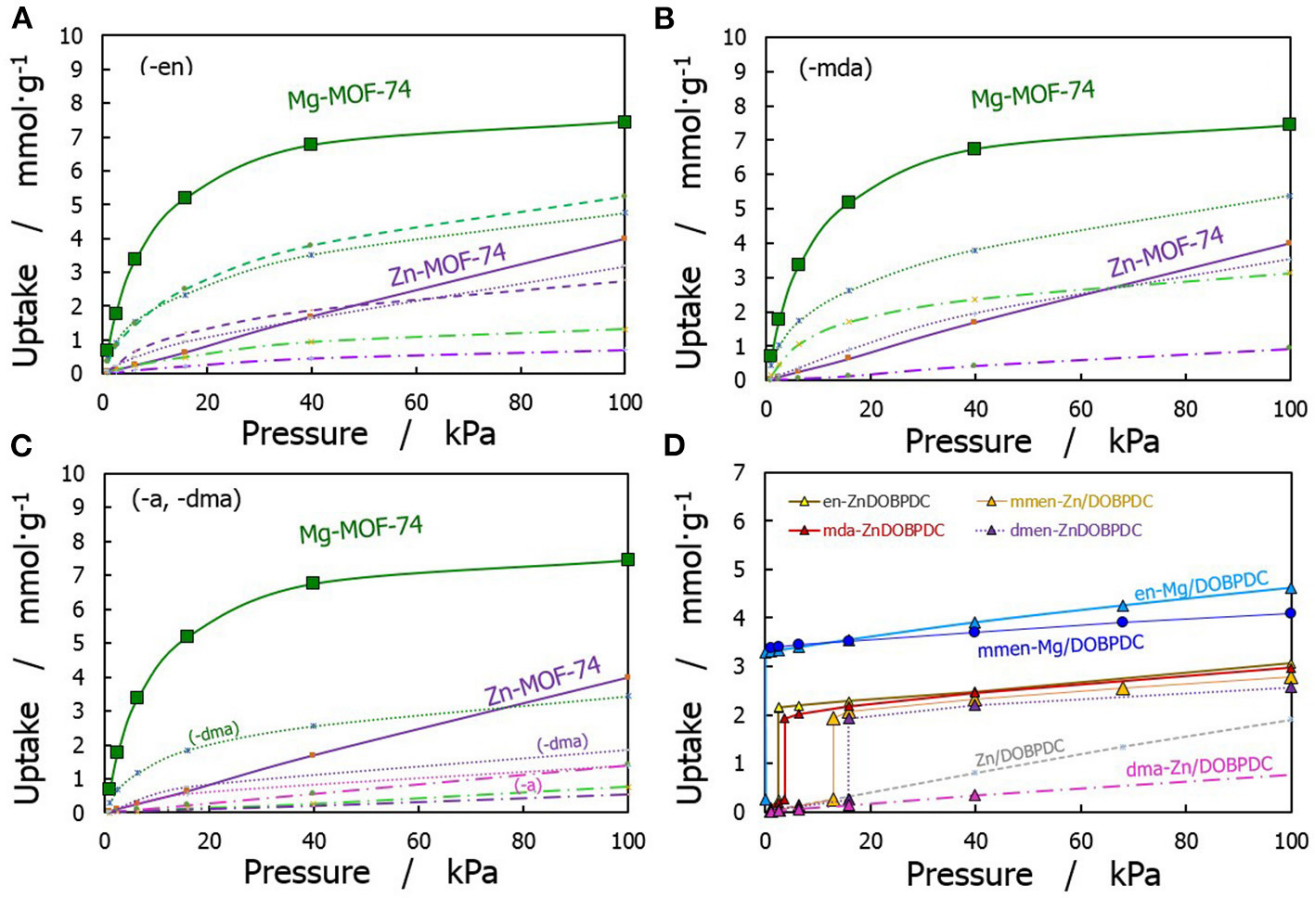
Comparison of simulated CO_2_ adsorption isotherms at 313 K. **(A)** MOF-74 functionalized with ethylenediamine, **(B)** MOF-74 functionalized with methanediamine, and **(C)** MOF-74 functionalized with dimethylamine and ammonia moieties (dashed lines for 16%, dotted lines for 50%, and long dash dotted lines for 100% functionalization); **(D)** M/DOBPDC structures functionalized with different amines.

A comparison of CO_2_ adsorption isotherms for all the studied structures at 313 K is displayed in Figure 4, with Figures 4A–C displaying the performance of bare and functionalized M-MOF-74 structures. Mg-MOF-74 represented with the scaled isotherm. Amine-grafted Mg/DOBPDC adsorbents (see Figure 4D) show significant capture capabilities at very low pressures, which are similar to the uptake for the narrower bare Mg-MOF-74 and greater than the observed in Zn-MOF-74. Note that not all the amine groups were explicitly linked to CO_2_ in the GCMC simulations to capture the experimentally observed isotherm step. It was found that the functionalization degree for Mg/DOBPDC and Zn/DOBPDC with the studied diamine-appended molecules is typically 72 and 61%, respectively.

At 15 kPa, the adsorption capacity of Mg-MOF-74 is 5.7 mmol·g^−1^, and 0.6 mmol·g^−1^ for Zn-MOF-74. The adsorption uptake for the functionalized Mg-MOF-74 structures decreases about 50–60% at this condition, mostly due to lower interactions between CO_2_ molecules and the highly attractive metallic centers. In contrast, some functionalized structures for Zn-MOF-74 show slightly higher values up to 0.9 mmol·g^−1^. However, saturation is reached at lower pressure due to the reduction of the available void fraction in the amine-containing frameworks. The degree of functionalization in MOF-74 structures show that amine substitution below 100% provide better performance than the fully substituted material, as already stated in the literature (Qiao et al., 2016). This is more noticeable as the size of the grafted amine increases. A compromise must be achieved between the increase in the CO_2_-adsorbent interactions and the loss of capacity due to steric effects.

As shown in Figure 4D, the capacities of the bare materials at typical flue gas conditions are reduced by using the expanded hexagonal version (M/DOBPDC), however a more interesting phenomena appears in the functionalized materials. For instance, -en and -mmen versions of Mg/DOBPDC present a capacity of ~3.6 mmol·g^−1^ at 15 kPa, 15% higher than the one achieved with the bare material (not shown). Furthermore, amine-functionalized versions of Zn/DOBPDC show a significant increase in the uptake at low pressures. The adsorption capacities at 15 kPa with Zn/ and Mg/DOBPDCs range from 2.1 to 3.5 mmol·g^−1^ respectively, higher than the representative amine-functionalized silica MCM-41-PEI-50 (Mason et al., 2015; Liao et al., 2016), with 1.5 mmol·g^−1^ under equivalent conditions. Hypothetically speaking, structures with an adsorption capacity of 5 mmol·g^−1^ can be achieved with the studied structures after 100% functionalization and carbamic acid formation in the expanded M/DOBPDC. However, no materials exhibiting more than 70–80% of functionalization have been experimentally synthesized yet for this MOF family (Sumida et al., 2011; Bernini et al., 2015).

According to the results presented in Figure 4D, the performance order for the adsorption uptake of the different studied amine functional groups was found to be –en, –mda > -mmen > -dmen. Two main reasons for this behavior are: -mda and –en have two active amine functional groups, which allow more specific interactions with CO_2_ molecules. The other reason is due to steric hindrance (Didas et al., 2012), since bigger and bulkier molecules reduce the CO_2_ accessibility.

### Selectivity and Temperature Swing Adsorption Process Evaluation

**Figure 5:**
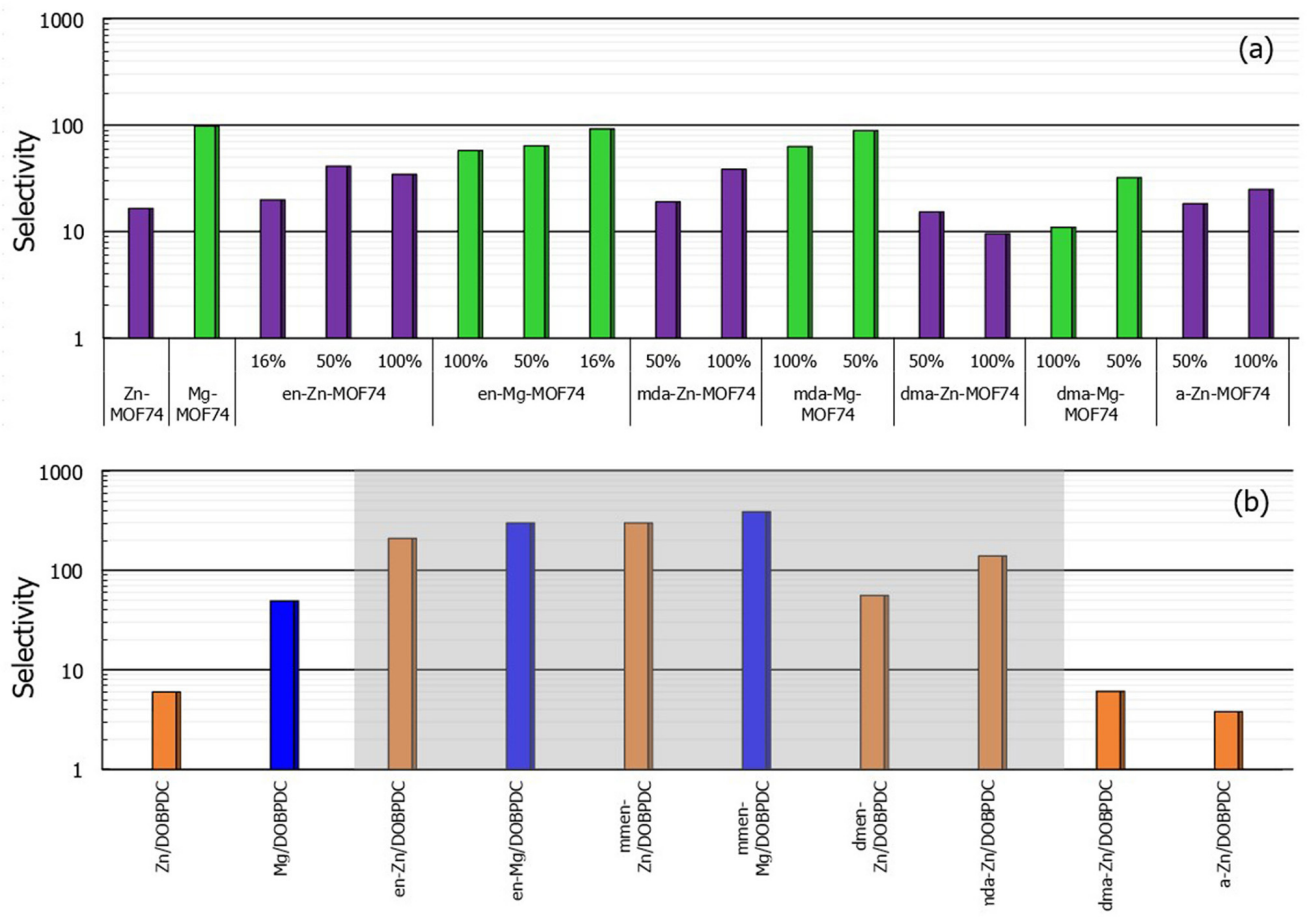
**(a,b)** Calculated selectivity values of binary 15% CO_2_ / 85% N_2_ mixtures (*T* = 313 K and *P*_total_ = 100 kPa) for M-MOF-74 and M/DOBPDC materials studied in this work (Color code: Mg-MOF-74 based structures in green, Zn-MOF-74 in violet, Mg/DOBPDC in blue and Zn/DOBPDC in orange). Percentages correspond to the degree of functionalization.

Figure 5 shows the simulated selectivity values from 15% CO_2_/85% N_2_ mixtures at 313 K and a total pressure of 100 kPa. Compared with the bare MOF-74, the functionalized versions with magnesium as metal center do not present enhancement on the CO_2_-over-N_2_ affinity. Conversely, some zinc structures such as en-Zn-MOF-74 and mda-Zn-MOF-74 tripled their selectivity values. Mg-MOF-74 has the highest carbon dioxide selectivity (i.e., 97) among the bare studied structures. However, the selectivity becomes exceptionally high when it comes to amine-grafted Mg/DOBPDC MOFs. Obtained values for the structures with explicit chemisorption (highlighted in gray in Figure 5) range from 200 up to 350, in good agreement with the reported values of ~230 for functionalized Mg/DOBPDC (Lee et al., 2014). Moreover, it should be noted that even the functionalized expanded hexagonal frameworks with zinc as metal center could reach such high values, making them potential materials to be explored for CO_2_ capture from flue gas since high purity can be achieved.

In a CO_2_ adsorption process, the adsorbent material should be regenerated after each CO_2_ uptake cycle. The most common method to regenerate the adsorbent is to recover CO_2_ by heating the adsorbent bed until full desorption is attained. Therefore, we calculated the working capacity related to temperature swing adsorption (TSA) cycles. The feed condition was taken as the binary 15% CO_2_/ 85% N_2_ mixture, and regeneration was set at 373 K. Since the amount adsorbed in bare materials gradually reduces as the temperature increases, these materials require a high desorption temperature to achieve large working capacities for separation. In the functionalized ones, with the explicit chemisorption analyzed in this study, it is observed that as the temperature increases the position of the isotherm step shifts to higher pressures (McDonald et al., 2015), allowing large working capacities and almost complete regenerability under specific conditions. However, using an adsorbent that binds CO_2_ too strongly would increase the temperature (energy) required to break the framework–CO_2_ interactions, hence making it less attractive from the regeneration point of view. Therefore, the adsorption step of these materials can be tuned by selecting the most promising amine balancing the conditions for the desired performance: the adsorption strength should be lower for the material to be readily regenerated, but not too low to strongly affect the selectivity and working capacity (Sumida et al., 2011, Bahamon et al., 2018).

**Figure 6:**
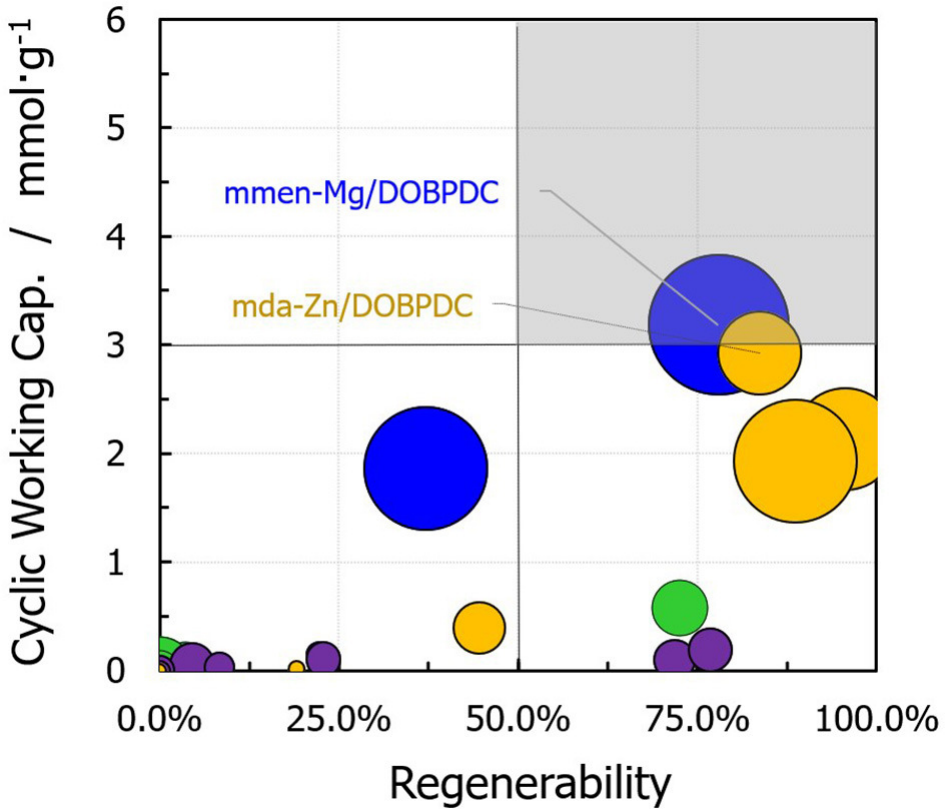
Carbon dioxide working capacity and regenerability values for functionalized structures under TSA conditions (*T*_regen_ = 373 K). The sizes of the circles represent the selectivity of the given material for CO_2_/N_2_ separation (Color code: same as in Figure 5).

Figure 6 shows a relationship between carbon dioxide regenerability (percentage) and the working capacity achieved, where the circle size represents the selectivity. The chart can be divided into four sections: MOFs grafted with primary diamines constitute the right bottom section (i.e., high adsorption at low CO_2_ pressures due to the strong interaction between the CO_2_ and the amine ends). Conversely, the left bottom section is mostly composed of the amine-functionalized M-MOF-74 structures, with a low cyclic working capacity due to reduced interactions between the heterodiamine and CO_2_. The right top section (highlighted in gray) contains mmen-Mg/DOBPDC, in which the methyl groups help in the facile desorption by weakening the Mg–carbamate interaction. Suitable adsorbents for a continuous CO_2_ capture TSA process should achieve the conditions of the highlighted section. In addition, mda-Zn/DOBPDC shows performance in the optimum range for post-combustion CO_2_ capture from flue gas, enabling a working capacity of 2.9 mmol·g^−1^ with a ΔT = 60°C for regeneration.

## Conclusions

In this study, GCMC molecular simulations were used to investigate the effect of different MOF amino-functionalized materials for carbon dioxide post-combustion capture by adsorption. A screening of synthetized and hypothetical materials, including different degrees of functionalization, was performed to identify key features of CO_2_ adsorbent materials for flue-gas applications.

The incorporation of amine groups grafted to the open metal sites of the MOFs can enhance the adsorption selectivity for CO_2_/N_2_ mixture separation. Results show that the functionalized expanded mmen-Mg/DOBPDC framework exhibits superior performance to their counterparts and the highest selectivities among the studied structures. Moreover, amine functionalization allows exceptionally high CO_2_ working capacity, and high regenerability, with potential promise for CO_2_ capture processes. mda-Zn/DOBPDC stands also as a promising material for such endeavor.

This study demonstrates that the CO_2_ adsorption properties of functionalized MOF materials can be tuned as a function of amine structures attached to the open metal sites, which can also be applied to other functionalized materials. Simulations have shown that the available void space left by the amine and the type of amine functional group plays a crucial role in achieving optimal CO_2_ capture properties, of great importance for the *ad-hoc* design of adsorbents using this feature. Further studies will include the performance evaluation of such adsorbent materials under more realistic industrial conditions, including trace impurities in the feed stream, and for Pressure/Vacuum Swing Adsorption processes with techno-economic assessment.

## Data Availability Statement

The raw data supporting the conclusions of this article will be made available by the authors, without undue reservation.

## Author Contributions

DB is a research scientist who carried out some of the GCMC simulations work, data analyses, interpretation, and original writing of the manuscript. WA was an undergraduate research assistant who performed some of the GCMC calculations for functionalized MOFs. MK and SB are assistant professors who helped in the selection of the systems, the employed methodology, and the writing of the manuscript. LV is a professor and the academic supervisor of the work, together with DB, she defined the strategy of the work, followed up on the results, interpretation and revised the overall work, and final version of the manuscript. All authors contributed to the article and approved the submitted version.

## Conflict of Interest

The authors declare that the research was conducted in the absence of any commercial or financial relationships that could be construed as a potential conflict of interest.
